# Effects of bromelain, melatonin, and thymoquinone on neutrophil extracellular trap formation and macrophage polarization in experimental periodontitis

**DOI:** 10.1590/1678-7765-2025-0699

**Published:** 2026-02-16

**Authors:** Şükran Acipinar, Mustafa Ozkaraca, Ufuk Okkay, Ali Sefa Mendil

**Affiliations:** 1 Sivas Cumhuriyet University Faculty of Dentistry Department of Periodontology Sivas Turkey Sivas Cumhuriyet University, Faculty of Dentistry, Department of Periodontology, Sivas, Turkey.; 2 Sivas Cumhuriyet University School of Veterinary Medicine Department of Pathology Sivas Turkey Sivas Cumhuriyet University, School of Veterinary Medicine, Department of Pathology, Sivas, Turkey.; 3 Ataturk University Faculty of Medicine Department of Medical Pharmacology Erzurum Turkey Ataturk University, Faculty of Medicine, Department of Medical Pharmacology, Erzurum, Turkey.; 4 Erciyes University Faculty of Veterinary Medicine Department of Pathology Kayseri Turkey Erciyes University, Faculty of Veterinary Medicine, Department of Pathology, Kayseri, Turkey.

**Keywords:** Adjuvants, Alveolar bone loss, Macrophages, Immunologic factors, Rats

## Abstract

Periodontitis is a chronic inflammatory disease characterized by dysregulated immune responses leading to alveolar bone loss. Formation of neutrophil extracellular traps and macrophage polarization play critical roles in its pathogenesis, and natural agents with immunomodulatory properties are being investigated as potential adjunctive therapies.

**Objective:**

This study aimed to compare the effects of systemically administered melatonin, bromelain, and thymoquinone on serum neutrophil extracellular trap formation and macrophage polarization in experimental periodontitis.

**Methodology:**

Forty rats were assigned to five groups: control, experimental periodontitis, and experimental periodontitis treated with melatonin, bromelain, or thymoquinone. Periodontitis was induced by ligature placement for 14 days. After ligature removal, the treatment groups received daily oral doses of melatonin, bromelain, or thymoquinone for 14 consecutive days. Biochemical, histologic, histomorphometric, and immunohistochemical analyses were performed. Comparisons between groups were performed using one-way ANOVA. Neutrophil extracellular trap levels measured before and after treatment within the same group were analyzed using a paired t-test.

**Results:**

Thymoquinone treatment significantly reduced alveolar bone loss compared to the bromelain and melatonin groups (p=0.00). The highest value was observed in the Periodontitis group and the lowest in the Thymoquinone group for neutrophil extracellular trap levels, with intergroup comparisons showing no statistically significant differences (p=0.597). All immunohistochemical analyses showed significant differences in all treatment groups compared to both the control and periodontitis groups (p=0.000). CD68(+) macrophages were most abundant in the periodontitis group and least in the Melatonin group (p=0.00). CD80 expression was highest in the Melatonin group and lowest in the Thymoquinone group (p=0.000), while CD206 expression was highest in the Bromelain group and lowest in the Thymoquinone group (p=0.00).

**Conclusion:**

Thymoquinone exhibited comparatively more favorable patterns, and overall, all agents showed trends that may support their consideration as adjunctive approaches in periodontitis management.

## Introduction

Periodontitis is a chronic inflammatory disease initiated by microorganisms within the dental biofilm and characterized by the progressive destruction of the tooth-supporting structures, including the alveolar bone.^1^ In susceptible individuals, the host mounts an immune-inflammatory response against the subgingival biofilm, which ultimately leads to irreversible periodontal tissue breakdown.

It is known that there is considerable interindividual variation in the inflammatory response to plaque accumulation, suggesting that host susceptibility is a critical determinant in disease development and pathogenesis.^2^ During inflammation, bacterial pathogens and their byproducts trigger immune responses, leading to the infiltration and activation of neutrophils, macrophages, plasma cells, and lymphocytes. This results in the release of inflammatory cytokines and enzymes, which contribute to the destruction of periodontal tissues.

Neutrophils are central to the acute periodontal inflammatory phase. They migrate to the site of infection and perform key antimicrobial functions such as phagocytosis, degranulation, and the release of neutrophil extracellular traps (NETs). NETs are web-like structures composed of DNA and antimicrobial proteins such as histones, neutrophil elastase, and lysozyme. These structures trap and neutralize bacteria, yet excessive or dysregulated NET formation has been associated with tissue damage and chronic inflammation.^3^ It has been shown that NET formation increases in periodontal lesions and that they play a role in the pathogenesis of periodontitis.^4^

Macrophages also play critical roles in both tissue homeostasis and immune defense. They are involved in bacterial clearance, antigen presentation, and immune regulation, as well as in maintaining balance between microbial load and host responses. By a process known as polarization, macrophages can adapt to different stimuli and tissue microenvironments by assuming distinct phenotypes. The two major phenotypes include M1-type (classically activated) macrophages, which exhibit pro-inflammatory and osteoclastogenic properties, and M2-type (alternatively activated) macrophages, which contribute to anti-inflammatory signaling, tissue repair, and regeneration. M1 macrophages cooperate with Th1 cells to eliminate pathogens and mediate bone resorption, while M2 macrophages support Th2 responses and facilitate healing.^5^

A dysregulated shift toward the M1 phenotype over the M2 phenotype is a contributing factor to periodontal destruction. M2 macrophages support the resolution of inflammation by promoting the apoptosis of polymorphonuclear leukocytes (PMNs) and M1 macrophages, releasing anti-inflammatory mediators and stimulating osteoblast activity. The balance between M1 and M2 polarization plays a critical role in transitioning from tissue breakdown to repair. Thus, macrophage polarization is increasingly recognized as a potential therapeutic target in the management of periodontal disease.

Standard treatment for periodontitis involves mechanical debridement, primarily scaling and root planing, to reduce microbial burden. However, such treatments may not fully prevent recolonization, and microbial resistance remains a concern. Although adjunctive antibiotic therapy can help modulate the host response, its use is limited due to adverse side effects. These challenges have spurred interest in plant-derived products that possess anti-inflammatory, antioxidant, and antimicrobial properties. Such compounds offer a low-risk adjunctive option to conventional therapy.^6^

Bromelain (BR) is a proteolytic enzyme extracted from Ananas comosus with documented anti-inflammatory, antioxidant, antithrombotic, and anticancer properties.^7,8^ It has been shown to inhibit periodontopathogen growth and reduce neutrophil chemotaxis and tissue destruction in the periodontal milieu.^9,10,11^ Given its safe pharmacological profile, BR is considered a promising therapeutic adjunct for periodontal disease.

Melatonin (ML) is an endogenously synthesized hormone that regulates circadian rhythms and exhibits potent anti-inflammatory, antioxidant, and immunomodulatory effects. ML is synthesized in different types of immune system cells, tissues, and organs, such as bone marrow, lymphocytes, leukocytes, and skin, but is mainly synthesized in the pineal gland. ML inhibits the synthesis of pro-inflammatory mediators, enhances the expression of anti-inflammatory cytokines, and regulates transcription factors related to inflammation.^12^In addition, ML is known to be an important agent in regulating macrophage phenotype. ML plays a role in regulating macrophage phenotypes by suppressing M1 polarization and promoting M2 polarization through STAT1/NF-κB/NLRP3 inhibition and STAT6 activation in macrophages.^13^

Thymoquinone (TQ), the primary bioactive component of Nigella sativa essential oil, possesses antioxidant, anti-inflammatory, antimicrobial, and anticancer properties.^14,15^With a low incidence of adverse effects,^16^ TQ has been explored as a therapeutic agent in various inflammatory diseases, especially periodontal disease.^17^

Despite substantial evidence on the systemic actions of BR, ML, and TQ, their comparative effects on macrophage polarization and NET formation in periodontitis remain largely unexplored. Considering the pivotal role of these immune pathways in periodontal pathology, this study aimed to address this gap by evaluating the impact of TQ, ML, and BR on macrophage polarization and NET formation in a rat model of ligature-induced periodontitis. We hypothesized that BR, ML, and TQ would attenuate periodontal inflammation by modulating macrophage polarization—reducing M1-associated pro-inflammatory signaling and promoting M2-associated resolution pathways—and by limiting excessive NET formation. By these combined immunoregulatory actions, we anticipated a subsequent mitigation of periodontal tissue destruction and alveolar bone loss.

## Methodology

The experimental protocol was approved by the Animal Ethics Committee of Cumhuriyet University (Approval No: 20.10.2023-65202830-050.04.04-770). Prior to this approval, institutional permission was obtained from the Sivas Cumhuriyet University Experimental Animal Research and Application Center. All procedures were conducted in accordance with relevant ARRIVE guidelines and ethical regulations. Forty male healthy Wistar rats (12 weeks old, weighing 220–250 g) were selected for this study. The rats were randomly divided into five groups using a simple manual method (drawing lots) (n=8 per group): control (C), experimental periodontitis (P), periodontitis treated with melatonin (M), periodontitis treated with bromelain (B), and periodontitis treated with thymoquinone (T). All procedures were conducted following the protocol approved by the Ethical Committee of the Faculty of Veterinary Sciences at Sivas Cumhuriyet University. Rats were housed in pairs in cages within a controlled environment maintained at a 12-hour light/dark cycle, temperature of 23°C ± 2°C, and humidity of 55% ± 10%. Food and water were provided ad libitum throughout the study.

The rats were anesthetized with intraperitoneal injections of xylazine (10 mg/kg) and ketamine (40 mg/kg) (Eczacıbaşı, İstanbul, Turkiye). Under anesthesia, experimental groups underwent ligation of the right lower first molars with sterile 4-0 silk sutures (Doğsan, İstanbul, Turkiye) placed submarginally for 14 days, following previously established protocols.^17^ Sutures were inspected daily, and any that became dislodged or loosened were promptly replaced.

BR, ML, and TQ were administered systemically via oral gavage at a dose of 10 mg/kg/day. Rats in the B, M, and T groups received their respective treatments—BR, ML, and TQ—for 14 consecutive days, following previously established protocols.^11,17,18^On day 14, the rats were sacrificed; mandibles were carefully dissected, with surrounding muscles and soft tissues removed while preserving the attached gingiva with the bone. The right mandibles were then used for histomorphometric and histologic analyses. All experimental procedures were carried out by two researchers (Ş.A., M.Ö.) who were blinded to the group allocations to minimize observer bias throughout the study.

### Group Allocation

Control group (C), n=8

Experimental periodontitis group (P), n=8

Experimental periodontitis + bromelain administration group (B), n=8 (10 mg/kg, 14 days; Sigma-Aldrich, Germany)

Experimental periodontitis + melatonin administration group (M), n=8 (10 mg/kg, 14 days; Sigma Chemical Co, USA)

Experimental periodontitis + thymoquinone administration group (T), n=8 (10 mg/kg, 14 days; Cayman, USA)

### Sample collection and biochemical assays

Before administration of BR, ML, and TQ (on day 14 of the experiment), rats from all groups were anesthetized, and blood samples were collected individually via cardiac puncture. Similarly, after 14 days of treatment (on day 28), rats in the B, M, and T groups were anesthetized for blood collection. The blood samples were centrifuged at 1500g for 10 min after collection. Serum samples were then stored at −80°C until analysis.

Serum NET activity was measured using a rat-specific enzyme-linked immunosorbent assay (ELISA) kit, following the manufacturer’s protocol (YLbiont, China, Catalog No: YLA1799RA).

### Histopathologic tissue preparation and analysis

The right mandibles were fixed in 10% neutral formaldehyde solution for histologic analysis. Following fixation, tissue samples were decalcified in 10% EDTA solution for three months and processed through a routine alcohol-xylol series. Five-micrometer-thick sections were stained with Hematoxylin and Eosin (H&E), and the distance between the cementoenamel junction (CEJ) and the bone crest (BC) as well as the gingival epithelium thickness were measured. All histologic measurements on H&E-stained 5-µm sections (CEJ–BC distance and gingival epithelium thickness) were independently evaluated by two blinded pathologists in six randomly selected fields per specimen.

### Immunohistochemical analysis and calculation of rankl, opg, cd68, cd80, cd206 positive cells

Paraffin-embedded tissue sections (5 µm) mounted on polylysine-coated slides were deparaffinized through a graded series of xylene and alcohol, rinsed with PBS, and incubated in 3% hydrogen peroxide (H₂O₂) for 10 minutes to inactivate endogenous peroxidase activity. Antigen retrieval was performed using a pepsin-containing solution at 37 °C for 10 minutes. After washing with PBS, the sections were incubated overnight at 4 °C with primary antibodies against RANKL (Affbiotech, Cat. No. AF0313) and OPG (Affbiotech, Cat. No. DF6824), each diluted 1:200.

Immunohistochemically, macrophage presence was assessed via CD68 expression, M1 macrophage activation via CD80, and M2 macrophage activation via CD206. Accordingly, sections were also incubated overnight at 4 °C with primary antibodies: CD68 (Elabscience, Cat. No. E-AB-70389), CD80 (Elabscience, Cat. No. E-AB-68320), and CD206 (Proteintech, Cat. No. 18704-1-AP), each at a 1:200 dilution.

A horseradish peroxidase (HRP)-conjugated secondary antibody (Thermo Fisher, Cat. No. TP-125-HL) was applied according to the manufacturer’s instructions. Visualization was achieved using 3,3’-diaminobenzidine (DAB) as the chromogen. Antibody validity was confirmed in our laboratory as follows: Positive control: A specific staining pattern consistent with known expression profiles was observed in rat spleen tissue, which is known to express the targeted antigens at high levels. Negative control: No specific signal was detected when the primary antibody was omitted, confirming that the staining was dependent on the presence of the primary antibody.

Following counterstaining with Mayer’s hematoxylin, sections were examined under light microscopy. Immunopositive cells were counted randomly in six different fields.

Both histopathological and immunohistochemical assessments were conducted using a Leica microscope (Leica, DM2500) equipped with an HC PLAN 10×/20 eyepiece. Under this optical configuration, the field of view diameter is approximately 2000 µm (≈ 3.14 mm^2^) when using the 10× objective lens, and approximately 1000 µm (≈ 0.785 mm^2^) when using the 20× objective lens.

### Statistical analysis

The sample size for each group (n=8) was determined based on a priori power analysis using G*Power version 3.1. The analysis was conducted to detect a medium effect size (f=0.25), with a power of 80% and an alpha level of 0.05, using one-way ANOVA. This sample size was deemed sufficient to identify statistically significant differences between the experimental groups with adequate power. The data were analyzed using GraphPad Prism 8 (GraphPad Software, USA). Normality of distribution was assessed using the Shapiro–Wilk test, which confirmed a normal distribution. Comparisons between groups were performed using one-way ANOVA followed by Tukey’s post hoc test. Independent t-tests were used for pairwise comparisons. NET levels measured before and after treatment within the same group were analyzed using a paired t-test. A p-value of <0.05 was considered statistically significant. Results are presented as mean ± standard deviation (SD) for each group.

Both histopathological and immunohistochemical evaluations were performed manually using the Cell Counter plugin of the ImageJ software. To assess the reproducibility of the counts, the same six histologic fields were independently evaluated by two different researchers. Inter-observer agreement was analyzed using the Intraclass Correlation Coefficient (ICC), calculated with a two-way random-effect absolute agreement model (ICC(2,1) = 0.73), indicating a good level of consistency between observers. Additionally, intra-observer reliability was confirmed by repeated counts, yielding a coefficient of variation (%CV) of 13.6%, thereby demonstrating the repeatability of the measurements.

## Results

### Serum NET levels

Before the induction of experimental periodontitis, NET levels were 3.27±0.68 ng/mL in the M group, 3.58±0.32 ng/mL in the B group, and 3.19±0.77 ng/mL in the T group. After periodontitis was induced, the level in the untreated P group was 4.05±2.37 ng/mL. Following 14 days of treatment, NET levels were 3.11±0.71 ng/mL in the M group, 2.95±0.91 ng/mL in the B group, and 2.69±0.54 ng/mL in the T group. The highest value was observed in the P group and the lowest in the T group. However, these differences were not statistically significant (p=0.597).

### Histopathological results

Histopathological examinations revealed statistically significant differences between the groups in terms of the CEJ–BC distance and gingival epithelial thickness (p=0.00). The shortest CEJ–BC distance was observed in the control C group (148.3±7.06 µm), while the greatest distance was found in the P group (347.5±6.07 µm). Among the treatment groups, the CEJ–BC distances were as follows: B group (240.5±10.41 µm), M group (200.9±6.35 µm), and T group (182.1±6.26 µm), from highest to lowest.

**Figure 1:**
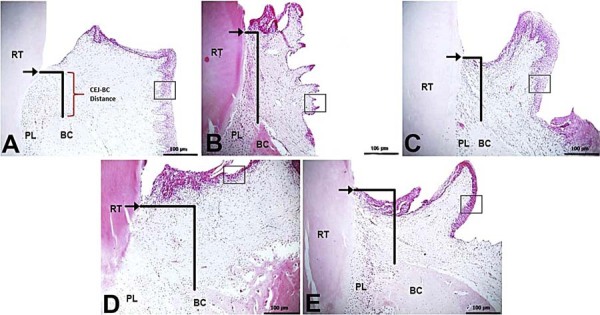
Histologic evaluation of alveolar bone loss and gingival epithelium thickness (H&E, x10).

For gingival epithelial thickness, the thinnest epithelium was measured in group P (2.75±1.28 µm). Compared to the C group (21.63±2.66 µm), the T group exhibited the thickest gingival epithelium (38.13±2.16 µm). The M group showed moderate epithelial thickness (15.75±1.66 µm), while the B group presented a mild increase (9.50±1.60 µm) (Figure 1).

A) C group. Normal CEJ-BC distance and gingival epithelial thickness; B) P group. Marked CEJ-BC distance and thin epithelial thickness; C) T group. Mild CEJ-BC distance and normal-to-increased epithelial thickness; D) B group. Moderate CEJ-BC distance and mild epithelial thickness; E) M group. Mild/moderate CEJ-BC distance and moderate epithelial thickness. RT: Root of Tooth, BC: Bone Crest, PL: Periodontal ligament, → (Cement-Enamel Junction), □ (Thickness of Gingival Epithelium).

### Immunohistochemical Results

**Figure 2:**
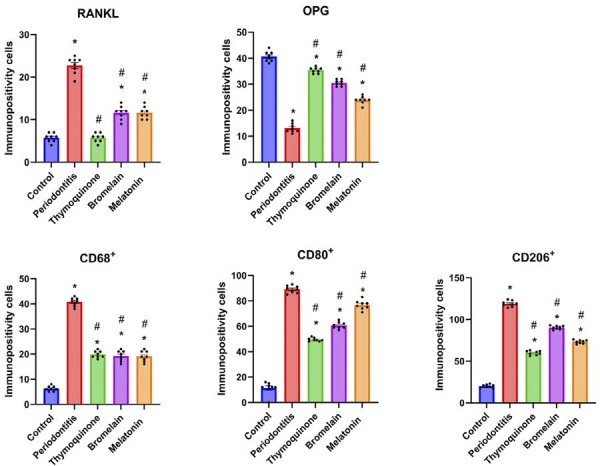
Statistical analysis of RANKL, OPG, CD68+, CD80+, CD206+ immunopositivity. Data are presented as mean±standard deviation. * indicates a significant difference compared to the control group (p<0.05), while # indicates a significant difference compared to the periodontitis group. (*, # p<0.05).

**Figure 3:**
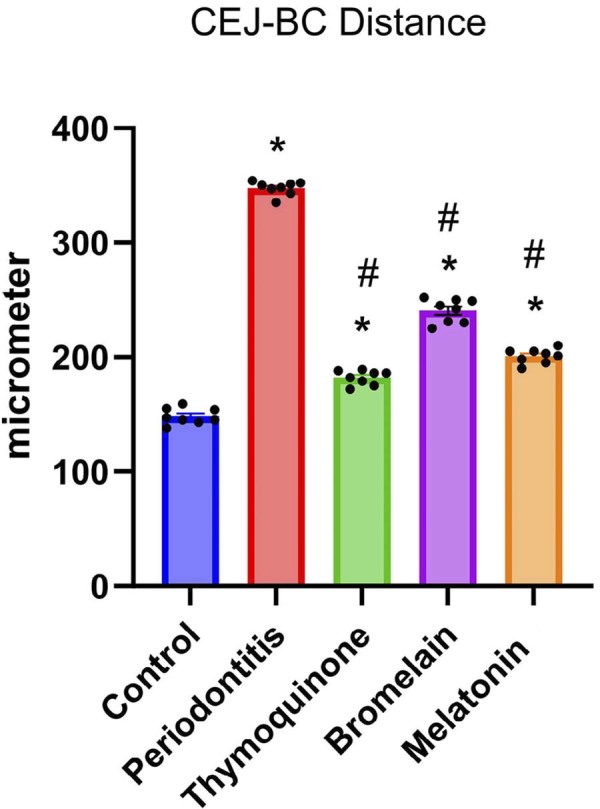
Statistical analysis of CEJ-BC distance. Data are presented as mean±standard deviation. * indicates a significant difference compared to the control group (p<0.05), while # indicates a significant difference compared to the periodontitis group. (*, # p< 0.05).

Immunohistochemical analyses for RANKL, OPG, CD68⁺, CD80⁺, and CD206⁺ demonstrated statistically significant differences between the groups (p=0.00, Figure 2). RANKL immunopositivity was highest in the P group (22.75±1.98), followed by moderate levels in the B (11.63±1.59) and M (11.63±1.40) groups, and mild levels in the C (5.75±1.03) and T (6.23±1.13) groups (Figure 3).

**Figure 4:**
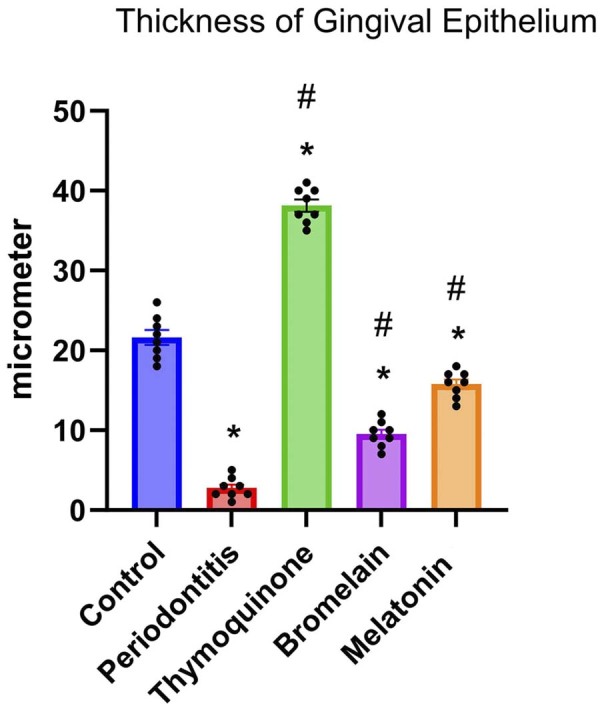
Statistical analysis of gingival epithelial thickness. Data are presented as mean±standard deviation. * indicates a significant difference compared to the control group (p<0.05), while # indicates a significant difference compared to the periodontitis group. (*, # p<0.05).

OPG immunopositivity was greatest in the C group (40.6±1.13) and lowest in the P group (13.13±1.72). Among the treatment groups, the T group (35.38±1.06) showed the highest OPG expression, approaching that of the control (p=0.00). The B (30.50±1.19) and M (23.88±1.45) groups also exhibited elevated OPG expression compared to the P group, though slightly reduced compared to the T group (Figure 4).

**Figure 5:**
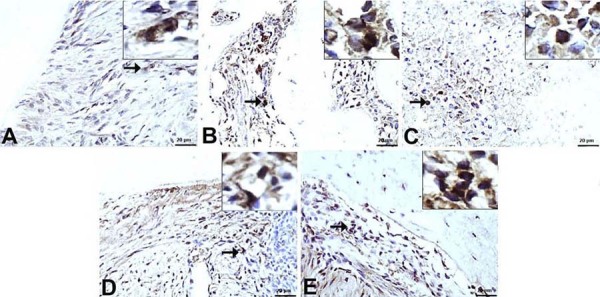
CD80+ immunopositivity in gingival tissues (IHC, x20). A) C group: Mild immunopositivity; B) P group: Marked immunopositivity; C) T group: Moderate immunopositivity; D) B group: Moderate to marked immunopositivity; E) M group: Marked immunopositivity. (→ indicates the area of interest).

The number of CD68⁺ macrophages was mild in group C (6.25 ±1.03), severe in group P (40.75±1.66), and moderate in the T (19.88±1.35), B (19.25±2.12), and M (19.13±2.10) groups. CD80⁺ (M1 phenotype) expression was highest in the P group (89.13±2.90), followed by the M (76.75±3.61), B (60.63±2.61), T (49.50±1.41), and C (11.38±4.89) groups (Figure 5) (p = 0.00).

**Figure 6:**
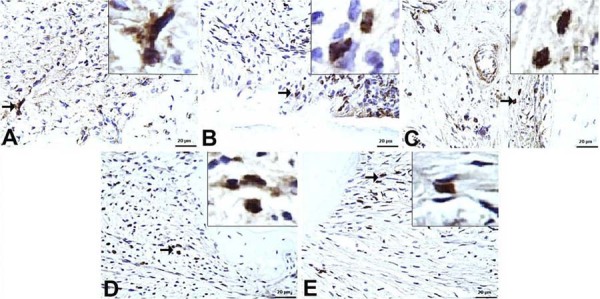
CD206+ immunopositivity in gingival tissues (IHC, x20). A) C group: Mild immunopositivity; B) P group: Marked immunopositivity; C) T group: Mild/moderate immunopositivity; D) B group: Marked immunopositivity; E) M group: Moderate immunopositivity. (→ indicates the area of interest).

Conversely, CD206⁺ (M2 phenotype) cell counts were lowest in the control group (20.00±2.07) and highest in the P group (118.80±3.53). Among the treatment groups, CD206⁺ expression was observed at 90.25±2.12 in the B group, 73.25±2.12 in the M group, and 59.88±2.53 in the T group (Figure 6) (p=0.00).

## Discussion

Osteoimmunology is an interdisciplinary field that explores the bidirectional interactions between the skeletal system and immune system cells. Its primary goal is to modulate the transition from a pro-inflammatory state to one of resolution and regeneration.^19^ The increasing recognition of osteoimmunology has led to a paradigm shift in research and clinical perspectives, emphasizing the roles of immune cells within bone tissue—particularly neutrophils and macrophages. These cells play a critical role in evaluating and regulating the processes of bone healing, repair, and regeneration.

Although BR, ML, and TQ are recognized for their antioxidant and anti-inflammatory properties, limited research has investigated their specific effects on macrophage polarization and NET formation in the pathogenesis of periodontitis.

In this study, we evaluated the effects of BR, ML, and TQ in experimental periodontitis. Among the treatment groups, the T group yielded the most favorable outcomes in terms of CEJ–BC distance and gingival epithelial thickness. Immunohistochemically, TQ again provided the best results by reducing CD80⁺ macrophages; conversely, it showed the lowest CD206 immunopositivity among all treatment groups, whereas the highest CD206 immunopositivity was observed in the B group. Serum NET levels decreased in all treatment groups compared to the P group; however, these differences were not statistically significant.

It is hypothesized that neutrophils and macrophages may serve as key targets for host modulation in the management of periodontal disease, due to their roles in resolving inflammation and initiating tissue repair.^20,21^Hyperactive neutrophils are well recognized as detrimental to periodontal tissues,^22^ and elevated NET formation has been associated with periodontal tissue destruction^4^. Furthermore, the presence of neutrophils and NETs has been confirmed in the dental biofilm.^23^ In this study, the highest NET levels were observed in the P group, while administration of BR, ML, and TQ led to a reduction in these levels. These findings are consistent with previous studies demonstrating the accumulation of NETs at sites of inflammation.^4,22^

Macrophages and osteoclasts have a common origin and a close relationship. To gain a comprehensive understanding of macrophages’ role in bone metabolism, it is essential to consider the cellular plasticity between these cell types. Macrophages have been shown to significantly influence osteoblast function, mineralization, and differentiation, and depletion of macrophages results in a 23-fold decrease in osteoblast differentiation and mineralization.^24^ The polarization and tissue-specific functions of macrophages, which range from promoting osteoblastogenesis to osteoclastogenesis, are likely to affect periodontal tissue destruction and potentially contribute to the pathogenesis of periodontitis.

M1 macrophages predominate during the initial phases of acute inflammation and possess the capacity to induce tissue damage. In contrast, the M2 phenotype plays a pivotal role in resolving inflammation, facilitating tissue repair and remodeling, and secreting anti-inflammatory cytokines to prevent further exacerbation of the inflammatory response. Researchers have focused on mitigating inflammation-induced damage and promoting tissue regeneration by increasing the M2/M1 macrophage ratio.^25^ For example, a study demonstrated that inhibition of M1 macrophages coupled with restoration of M2 macrophages reduced alveolar bone loss in diabetic rat models.^26^ Polarization towards the M2 phenotype is known to promote osteogenic differentiation. In our study, macrophage distribution was highest in the P group across all phenotypes. The lowest levels of M1 macrophages were observed in group C, followed by groups T, B, and M, with the highest levels in group P. Conversely, the lowest levels of M2 macrophages were detected in group C, followed by T, M, B, and P groups, respectively. Given that the inflammatory state was not triggered, it was expected that both M1 and M2 macrophages would exhibit low levels of expression in Group C. The relatively short, 14-day experimental periodontitis model may explain why M2 macrophage levels remained elevated in the P group, as M2 macrophages are known to secrete proinflammatory cytokines and chemokines at early stages as part of the host defense mechanism.^27^

BR exhibits anti-inflammatory, antioxidant, and antibacterial properties against periodontopathogens and has been identified as a potential adjunct in the non-surgical management of periodontitis.^9,10^A study demonstrated that BR effectively reduces neutrophil migration to sites of inflammation.^10^ Furthermore, a recent clinical trial on pocket elimination surgery reported that postoperative administration of BR significantly decreased bleeding on probing compared to placebo.^28^ BR has also shown efficacy in controlling pain following periodontal surgery.^29^ Consistent with these findings, this study observed that BR reduced alveolar bone loss and NET formation.

In studies on periodontitis, ML has been shown to reduce the RANKL/OPG ratio, increase total antioxidant capacity, and limit periodontal tissue destruction.^30,31^ Maria and Witt-Enderby^32^ reported that ML promotes osteoblast proliferation. A recent clinical study demonstrated that systemic ML administration did not affect OPG levels but decreased RANKL and MMP-8 levels, resulting in improved clinical outcomes.^33^ Interestingly, ML’s effects on NET formation seem to vary: in a polymicrobial infection model, ML promoted NET development, whereas in a recent study on lung injury, it suppressed NET formation.^34,35^ This dual role of NETs may be explained by their function in both suppressing inflammation and supporting immune responses, depending on the severity and pathogenesis of the inflammatory condition. Given these properties, ML has the potential to influence bone tissue remodeling. Consistent with these findings, our study has found that ML treatment reduced alveolar bone loss and NET formation.

In this study, TQ demonstrated a significant reduction in alveolar bone loss and RANKL expression compared to BR and ML. Additionally, TQ treatment resulted in a greater increase in gingival thickness than the C group. While TQ reduced M1 macrophage levels more effectively than BR and ML, its ability to shift macrophage polarization toward the M2 phenotype was comparatively limited. The effect of TQ on M1 macrophages can be attributed to its potent anti-inflammatory property by targeting multiple signaling pathways. The limited effect on M2 macrophages can be attributed to the short duration of administration. Wilson, et al.^36^ (2015) reported that short-term treatment of TQ suppressed the M2 phenotype. Consistent with these findings, Ozdemir, et al.^17^ (2012) reported that TQ attenuates alveolar bone loss and downregulates proinflammatory cytokine expression in an experimental periodontitis model. Furthermore, Tantivitayakul, et al.^37^ (2020) found that TQ reduces the virulence of periodontopathogens Fusobacterium nucleatum and Porphyromonas gingivalis, suggesting its potential application in periodontal therapy to inhibit disease progression. The acceleration of epithelial proliferation by TQ, observed in our study, aligns with previous results.^38^ Baştuğ, et al.^39^ (2022) also demonstrated that TQ treatment significantly increased new bone formation, osteoblast numbers, and capillary density compared to controls. Although there are no studies specifically addressing the effect of TQ on NET formation, a meta-analysis of animal models indicated that Nigella sativa does not significantly impact cellular immunity, including neutrophil function.^40^

To date, no studies have directly compared the effects of BR, ML, and TQ on macrophage polarization and NET formation. In our study, NET formation did not differ significantly between the groups; however, all treatment agents resulted in a reduction compared to the P group. All agents also produced significant improvements in CEJ–BC distance and epithelial thickness compared to both the control and periodontitis groups. Among the treatments, thymoquinone presented the greatest benefits, followed by melatonin.

Furthermore, our findings indicate that BR, ML, and TQ may help reduce inflammation by influencing the host immune response in periodontitis, suggesting a potential role in supporting the balance between immune regulation and bone homeostasis. In particular, the pronounced effect of TQ on limiting alveolar bone loss and enhancing epithelial thickness is noteworthy.

In our study, periodontitis was induced with a 14-day ligature model. While inductions of up to 28 days have been used in studies investigating longer-term systemic effects in the literature, a 14-day period is preferred for alveolar bone loss and periodontal inflammation.^11^ A key strength of our study is the comprehensive analysis of these agents’ effects through histopathological, immunohistochemical, and biochemical methods within a well-established ligature-induced experimental periodontitis model. This model reliably mimics human periodontitis driven by microbial plaque accumulation. Nonetheless, as an animal study, it has inherent limitations. Therefore, our findings require validation by further studies that investigate other cellular components involved in the pathogenesis of periodontitis, include detailed assessment of the furcation region, and comprehensively examine the potential systemic effects of these agents, to better inform prevention and treatment strategies.

## Conclusions

As our understanding of osteoimmunology has advanced, the need to regulate excessive or dysregulated host responses in periodontal disease has become increasingly evident. This study demonstrates that bromelain, melatonin, and particularly thymoquinone have the potential to modulate key mechanisms involved in the pathogenesis of periodontitis. Our findings indicate that thymoquinone may positively influence the disease process both at the tissue level and by its effects on inflammatory response pathways. These findings provide new insights and strategies for the development of herbal products with osteoimmune regulatory effects.
